# Cost and outcomes of side-to-side versus end-to-end anastomosis in right colectomy: a retrospective cohort study

**DOI:** 10.1007/s00464-025-11544-4

**Published:** 2025-01-24

**Authors:** Kim Herzog, Stephanie Taha-Mehlitz, Kris Denhaerynck, Daniel C. Steinemann, Marc-Olivier Guenin, Marco von Strauss und Torney

**Affiliations:** 1 Clarunis University Digestive Health Care Center Basel, Basel, Switzerland; 2 Department of Public Health, University of Basel, Basel, Switzerland; 3 Clarunis Viszeralchirurgie, Kleinriehenstrasse 30, 4058 Basel, Switzerland

**Keywords:** Ileocecal anastomosis, Right colectomy, Ileocecal resection, Economy, Costs

## Abstract

**Background:**

The optimal anastomotic configuration for right-sided colectomies remains controversial, with comparable postoperative outcomes across techniques. Thus, economic considerations may play a larger role in decision-making within cost-constrained healthcare settings.

**Methods:**

This retrospective cohort study evaluated right-sided colectomies with ileocolic anastomosis at a Swiss tertiary center between 2016 and 2021. We compared costs and outcomes among three anastomotic techniques: end-to-end (EE), side-to-side stapled (SSS), and side-to-side hand-sewn (SSH).

**Results:**

Out of 468 patients (mean age 67.7 ± 16.2 years; 51.7% female), EE was used in 95 cases (20.3%), SSS in 191 (40.8%), and SSH in 182 (38.9%). The majority (72.0%) underwent elective surgery. Insurance coverage included basic (62.2%), semi-private (24.2%), and private (13.7%). Mean operation times were shortest for EE (173.6 ± 72.3 min), followed by SSS (188.0 ± 65.4 min) and SSH (195.1 ± 61.5 min). The median total costs were 26,449 €. The SSS group had the lowest median total costs, 2424 € less than SSH and 2095 € less than EE, with no significant differences (*p* = 0.1657). Significant differences were observed in operating room (OR) costs, with EE being the lowest and SSH the highest (*p* < 0.0001). Adjusted OR costs in SSH were 23% more than EE and 21% more than SSS. No significant difference was found between EE and SSS OR costs. For mean OR material costs, EE had the lowest, followed by SSS and SSH. Adjusted costs for EE were 52% lower than SSS (*p* = 0.0005) and 65% lower than SSH (*p* = 0.0191).

Clavien-Dindo Grade ≥ III complication rates were 2.1% for EE, 1.9% for SSH, and 3.0% for SSS. Anastomotic leakage occurred in 12 cases (2.6%), and in-hospital mortality was 0.9% (no deaths in EE; two in SSS and SSH each).

**Conclusions:**

EE anastomosis was associated with the lowest OR and material costs in this retrospective analysis while no disadvantages concerning postoperative outcomes could be identified.

Right-sided colectomy, a common surgical procedure for benign and malignant colorectal diseases, requires precise and effective anastomotic techniques to ensure optimal patient outcomes. Despite the high prevalence of this surgery, a gold standard has yet to be established for the anastomotic technique to restore intestinal continuity between the ileum and colon [1]. Various methods have been employed, including side-to-side or end-to-end configurations and hand-sewn or stapled anastomoses. Yet, comparative studies have failed to demonstrate a definitive advantage of one technique over the others [2–4]. This lack of differentiation pertains to several critical factors such as surgical or oncological outcomes, postoperative complications, and functional recovery [2, 5, 6].

One of the most significant postoperative concerns is anastomotic leakage, a complication with serious implications for patient recovery and an increase in readmission rates [6–8]. However, current research does not indicate a clear superiority of any specific anastomotic technique in reducing the incidence of this feared complication [5, 9, 10]. Similarly, assessments of functional outcomes and oncological outcomes have not revealed substantial differences among the different anastomotic methods [5, 11].

In light of the inconclusive evidence regarding the optimal anastomotic technique in relation to safety, it becomes imperative to consider other factors that might influence the choice of method. In the context of increasingly cost-constrained healthcare settings, the financial implications of each technique warrant closer examination [12–14]. Understanding the cost-effectiveness of the various configurations and suture materials used for anastomoses could provide valuable insights for decision-making, potentially guiding surgeons towards more economically viable practices without compromising patient care quality.

This study aims to explore the cost dynamics of the different anastomotic techniques in right-sided colectomies.

## Patients and methods

### Data collection & inclusion criteria

The study was a retrospective single-center cohort study of a Swiss tertiary referral center. Data were extracted retrospectively from the patient files of the hospital information system. Patients aged above 18 years who underwent right-sided colectomy or ileocecal resection with ileocolic anastomosis for all indications between January 1st, 2016, and December 31st, 2021, were eligible. Only data from patients who signed the general informed consent were included. Three types of anastomoses were compared: end-to-end (EE), side-to-side stapled (SSS), and side-to-side hand-sewn (SSH). The type of anastomotic formation was under the discretion of the operating surgeon and mainly influenced by surgeons’ preference. In rare occasions the bowel configuration (such as a distended colonic end compared to a small ileal stump) might have led to a preference towards SS configuration.

This retrospective cohort study was completed based on Strengthening the Reporting of Observational Studies in Epidemiology (STROBE) guidelines [15].

### Exclusion criteria

We excluded patients if the colectomy was an addition to another leading surgical procedure (e.g., multivisceral resections or gynaeco-oncological cases) or if a diverting ostomy was formed. Since up to the time of end of the data collection only 13 cases accounted for robotic-assisted procedures, we also excluded the latter.

### Endpoints

The *primary endpoint* was overall costs. These included all costs during the hospital stay. In subgroup analyses, operating room (OR) costs include all expenses incurred through the use of the operating room. OR material and medication costs were evaluated as costs for the material used in OR, excluding anesthesia. Costs were expressed in Euros using the average exchange rate during the period between 2016 and 2021 at a rate of 1 Euro = 0.9072 Swiss Francs [16], and corrected for yearly consumer index inflation [17].

The *secondary endpoint* was morbidity and 30-day mortality. Postoperative complications were classified using the Clavien-Dindo Classification (CD) and divided into two groups based on the necessity for an additional intervention (CD ≥ 3 and CD < 3). Special interest was given to anastomotic leakage.

### Variables and definitions

Demographic data on age, sex, body mass index (BMI), and American Society of Anesthesiologists (ASA) score were collected. Additionally, information on whether the procedure was elective or emergency, the etiology (oncological, bowel obstruction, inflammatory bowel disease (IBD), infection, other), length of stay (LOS) in days, operative time, intensive care unit stay (hours), surgeon’s experience (trainee, general surgeon, specialized visceral surgeon), teaching procedure (yes or no), and type of surgical approach (open, minimally invasive), anastomotic configuration, and insurance status were analyzed. The insurance status was categorized as either basic care on a compulsory basis or at a semi-private/private level.

Overall costs and reimbursement of the cases, in addition to surgical outcomes, were compared between the following types of anastomotic configurations: end-to-end (EE) and side-to-side (SS). Side-to-side anastomosis was further subdivided into stapled (SSS) and hand-sewn (SSH).

### Statistical analysis

Next to providing descriptive analyses for all variables involved, we compared the three types of anastomotic configurations with regard to sample characteristics and outcome variables using Kruskal–Wallis and Fisher’s exact tests. We contrasted logarithmically transformed costs between the treatment groups using linear mixed modeling, in which a random intercept compensated for a possible cost clustering between surgeons, and in which we adjusted for propensity scores that reflected possible confounding differences between the treatment groups. The propensity scores were obtained using multinomial logistic regression analysis by regressing the treatment groups onto the following confounding variable set: surgeon`s expertise, insurance status, approach, ASA score, sex, body mass index, teaching, surgery, age, length of stay ICU [18].

We considered the level of statistical significance at an alpha of 0.05, and executed the calculations in SAS 9.4 (SAS Institute Inc., NC).

### Patient and public involvement

Patients and the public have not been involved in planning, managing, designing or carrying out the research. The study was approved by the Northwestern and Central Ethics Committee Switzerland (BASEC-Nr 2022-01233).

## Results

During the study period, a total of 468 patients were included. In 95 (20.3%) cases, an EE was performed, while SSS was used in 191 (40.8%) cases and SSH in 182 (38.9%) cases.

### Demographics

The mean age was 67.7 ± 16.16 years and 51.7% were female. The preoperative physical status was classified using the ASA score with a median score of 3 (2–3). Regarding these variables between the three groups, there was no significant difference observed. The mean BMI was 24.68 ± 4.80 kg/m^2^ and was in EE the lowest at 23.75 ± 4.10 (*p* = 0.027). Overall, 62.2% of the patients had basic insurance followed by 24.2% semi-private and 13.7% private insurance. The highest rate of private insurance was observed in the EE group (*p* = 0.003). Further details for demographic data are shown in Table 1.

**Table 1:** Demographics

	Missings (*N*,%)	Total	EE	SSH	SSS	*p-value*
*N* (%)	0 (0%)	468 (100.0%)	95 (100.0%)	182 (100.0%)	191 (100.0%)	
Sex (*N*, %)	0 (0%)					
Male		226 (48.3%)	37 (38.9%)	92 (50.5%)	97 (50.8%)	0.1256
Female		242 (51.7%)	58 (61.1%)	90 (49.5%)	94 (49.2%)	
Age (years)	0 (0%)					
Mean (STD)		67.72 ± 16.16	69.36 ± 18.19	68.11 ± 14.60	66.53 ± 16.51	0.1902
Body mass index (kg/m^2^)	0 (0%)					
Mean (STD)		24.68 ± 4.80	23.75 ± 4.10	24.53 ± 5.07	25.28 ± 4.78	0.0268
ASA-score	8 (1.7%)					
I		13	4	2	7	0.3411
II		217	37	87	93	
III		202	48	76	78	
IV		28	6	12	10	
Median (IQR)		2.5 (2.0–3.0)	3 (2–3)	2 (2–3)	2 (2–3)	
Insurance status (*N*, %)	0 (0%)					
Basic insurance		291 (62.2%)	44 (46.3%)	113 (62.1%)	134 (70.2%)	0.0028
Semi-private		113 (24.2%)	32 (33.7%)	42 (23.1%)	39 (20.4%)	
Private		64 (13.7%)	19 (20.0%)	27 (14.8%)	18 (9.4%)	

*EE* end-to-end anastomosis, *SSH* side-to-side handsewn anastomosis, *SSS* side-to-side stapled anastomosis, *STD* standard deviation, *ASA* American Society of Anesthesiologists, *IQR* interquartile range

### Surgical procedure

Elective surgery was performed in 72.0% while 129 patients underwent emergency surgery. Two patients were referred from other institutions without significant alterations. The most frequent type of resection was right colectomy at 77.1%. A minimally invasive approach was chosen in 55.8% of cases, with a conversion rate of 8.8%. The use of SS anastomotic configuration was associated with a minimal-invasive approach compared to EE anastomosis (*p* = 0.0002). Minimal-invasive intracorporal anastomosis was not yet performed during the study period as the technique was not introduced in the institution up to this time. The mean overall operation time was 187.84 ± 65.73 min, with the SSH group taking the longest, followed by SSH and EE (*p* = 0.0012). The mean blood loss was 71.52 ± 109.38 ml with no significant differences between groups. In total, 138 cases were teaching operations, accounting for most of the cases (13.9%) in the SSH group (see Table 2).

**Table 2:** Surgical intervention

	Missings (*N*, %)	Total	EE	SSH	SSS	*p-value*
*N* (%)	0, 0%	468 (100.0%)	95 (100%)	182 (100%)	191 (100%)	
Surgery (*N*, %)	0, 0%					
Elective		337 (72.0%)	63 (66.3%)	132 (72.5%)	142 (74.3%)	0.5450
Emergency		129 (27.6%)	32 (33.7%)	49 (26.9%)	48 (25.1%)	
Referral		2 (0.4%)	0 (0.0%)	1 (0.5%)	1 (0.5%)	
Aetiology (*N*, %)	0, 0%					
Oncological		334 (71.4%)	74 (77.9%)	127 (69.8%)	133 (69.6%)	0.5482
Bowel obstruction		22 (4.7%)	4 (4.2%)	11 (6.0%)	7 (3.7%)	
IBD		32 (6.8%)	6 (6.3%)	11 (6.0%)	15 (7.9%)	
Infection		27 (5.8%)	2 (2.1%)	10 (5.5%)	15 (7.9%)	
Others		54 (11.5%)	9 (9.5%)	24 (13.2%)	21 (11.0%)	
Type of surgery (*N*, %)	0, 0%					
Extended right colectomy		26 (5.6%)	5 (5.3%)	8 (4.4%)	13 (6.8%)	0.5786
Right colectomy		361 (77.1%)	75 (78.9%)	146 (80.2%)	140 (73.3%)	
Ileocecal resection		81 (17.3%)	15 (15.8%)	28 (15.4%)	38 (19.9%)	
Approach (*N*, %)	0, 0%					
Minimal-invasive		261 (55.8%)	36 (37.9%)	104 (57.1%)	121 (63.4%)	0.0002
Open		207 (44.2%)	59 (62.1%)	78 (42.9%)	70 (36.6%)	
Conversion rate (*N*, %)	0, 0%	41 (8.8%)	5 (5.3%)	11 (6.0%)	25 (13.1%)	
Operation time (minutes)						
Mean (STD)		187.84 ± 65.73	173.57 ± 72.33	195.13 ± 61.48	187.99 ± 65.40	0.0017
Blood loss (ml)						
*N*	62 (13.3%)	406 (86.75%)	64 (67.4%)	161 (88.5%)	181 (94.8%)	
Mean (STD)		71.52 ± 109.38	86.80 ± 147.72	64.55 ± 72.39	72.32 ± 120.39	0.8497
Surgeon`s expertise	0, 0%					
Trainee		10 (2.1%)	5 (5.3%)	1 (0.5%)	4 (2.1%)	< 0.0001
General surgeon		104 (22.2%)	5 (5.3%)	39 (21.4%)	60 (31.4%)	
Specialized visceral surgeon		354 (75.6%)	85 (89.5%)	142 (78.0%)	127 (66.5%)	
Teaching (*N*, %)	0, 0%					
Yes		138 (29.5%)	17 (17.9%)	65 (35.7%)	56 (29.3%)	0.0075
No		330 (70.5%)	78 (82.1%)	117 (64.3%)	135 (70.7%)	
Supervised surgeon`s expertise in teaching procedures	0, 0%					
Trainee		87 (18.9%)	14 (14.7%)	34 (18.7%)	39 (20.4%)	0.1262
General surgeon		41 (8.8%)	2 (2.1%)	25 (13.7%)	14 (7.3%)	
Specialized visceral surgeon		10 (2.1%)	1 (1.1%)	6 (3.3%)	3 (1.6%)	

*EE* end-to-end anastomosis, *SSH* side-to-side handsewn anastomosis, *SSS* side-to-side stapled anastomosis, *IBD* inflammatory bowel disease, *STD* standard deviation

### Postoperative outcome

The overall median length of stay was 13 (10–18) days with the longest hospital stay of 14 (11–20) days in the EE group (*p* = 0.0095). Prior to the implementation of an Enhanced Recovery After Surgery (ERAS) program in 2019 the median LOS was 13 (10–19) days, whereas afterwards, the median LOS was 11 (9–16) days. In 25 cases, a major postoperative complication (Clavien-Dindo ≥ 3) occurred with no significant differences between groups (*p* = 0.1900) as well as case-related complications (*p* = 0.5032). With 9 cases (1.9%), the majority of major postoperative complications were anastomotic leaks followed by anastomotic bleeding (4 cases, 0.85%) and abdominal wound dehiscence (4 cases, 0.85%) and others. Four patients died within 30 days after surgery. Of these, two cases were emergencies caused by advanced cancer and the therapy was discontinued after multiorgan failure. One patient suffered cardiac arrest directly after surgery and the other patient died because of ileus and aspiration. Regarding anastomotic leaks, we observed in total 12 (2.6%) anastomotic leaks, 2 in the EE group, 6 in the SSH group and 4 in the SS group (*p* = 0.7477). In total 9 patients were in need for interventional treatment, either surgery or drainage, the other 3 patients could be treated with antibiotics only. The postoperative outcomes are summarized in Table 3.

**Table 3:** Outcome

	Total	EE	SSH	SSS	*p-value*
*N* (%)	468 (100.0%)	95 (100.0%)	182 (100.0%)	191 (100.0%)	
Length of hospital stay (days)					
Median (IQR)	12.5 (10–18)	14 (11–20)	11.5 (9–16)	12 (12–18)	0.0095
Length of stay ICU (hours)					
Median (IQR)	0 (0–19)	0 (0–20)	0 (0–19)	0 (0–19)	0.9968
Complications postoperative (Clavien/Dindo)					
< 3 (minor)	17 (3.6%)	1 (1.1%)	7 (3.8%)	9 (4.7%)	0.1900
≥ 3 (major)	25 (5.3%)	2 (2.1%)	9 (4.9%)	14 (7.3%)	
None	426 (91.0%)	92 (96.8%)	166 (91.2%)	168 (88.0%)	
Complications case-related (Clavien/Dindo)					
< 3	76 (16.2%)	12 (12.6%)	31 (17.0%)	33 (17.3%)	0.5032
≥ 3	20 (4.3%)	2 (2.1%)	7 (3.8%)	11 (5.8%)	
None	372 (79.5%)	81 (85.3%)	144 (79.1%)	147 (77.0%)	
Anastomotic leaks (*N*, %)	12 (2.6%)	2 (2.1%)	6 (3.3%)	4 (2.1%)	0.7477
30 day mortality (*N*, %)	4 (0.8%)	0 (0.0%)	2 (1.1%)	2 (1.0%)	0.8410

*EE* end-to-end anastomosis, *SSH* side-to-side handsewn anastomosis, *SSS* side-to-side stapled anastomosis, *IQR* interquartile range, *ICU* intensive care unit

### Costs

The median overall total costs were 26,449 €, as shown in Table 4. The SSS group had the lowest mean total costs and was 2424 € cheaper than SSH and 2095 € cheaper than EE with no significant differences. Significant variations were observed in OR costs, with the lowest in EE and the highest in SSH (*p* < 0.0001). Adjusted average OR costs in SSH were 23% (= 1/0.91, Table 5) more expensive than in EE, and 21% more expensive than SSS. The difference between the OR costs in EE and SSS was not significant. Similar trends were found in OR material costs. The EE group had the lowest costs, averaging 1681 ± 2456 € followed by the SSS group at 2547 ± 1312 €, and the SSH group at 2833 ± 1737 € (*p* < 0.0001). Adjusted average costs for the EE group were about half of SSS costs (52%; *p* = 0.0005) and two thirds of the SSH group (65%; *p* = 0.0191) for OR materials.

**Table 4:** Costs (€)

	Total	EE	SSH	SSS	*p-value*
Total costs (expenses)					
*N* (%)	468 (100%)	95 (100.0%)	182 (100.0%)	191 (100.0%)	0.1657
Mean (STD)	31,842 ± 18,675)	31,478 ± 17,936)	32,805 ± 19,650	31,104 ± 18,129	
Median (IQR)	26,449 (15,295)	27,391 (16,467)	27,720 (14,176)	25,296 (16,689)	
OR costs					
*N* (%)	468 (100%)	95 (100.0%)	182 (100.0%)	191 (100.0%)	< 0.0001
Mean (STD)	3964 ± 2026	3488 ± 1325	4456 ± 2269	3733 ± 1978	
Median (IQR)	3712 (1576)	3402 (1316)	4027 (1586)	3528 (1845)	
OR material costs					
*N* (%)	464 (99.1%)	95 (100.0%)	179 (98.9%)	190 (99.5%)	< 0.0001
Mean (STD)	2480 ± 1808	1681 ± 2456	2833 ± 1737	2547 ± 1312	
Median (IQR)	2501 (1877)	1255 (1550)	2962 (1343)	2558 (1737)	

*EE* end-to-end anastomosis, *SSH* side-to-side handsewn anastomosis, *SSS* side-to-side stapled anastomosis, *STD* standard deviation, *IQR* interquartile range, *OR* operating room

**Table 5:** Propensity score-adjusted estimations of cost differences between the surgical procedures

Outcome variable	Estimates (95% CI)		*p-value*
	Estimates of logarithmized outcome variables	Exponentiated estimates	
Total costs (expenses)			
EE versus SSS (reference)	−0.1002 (−0.2428 − 0.0424)	0.90 (0.78 − 1.04)	0.1678
EE versus SSH (reference)	−0.1095 (−0.2487 − 0.0296)	0.90 (0.78 − 1.03)	0.1224
SSH versus SSS (reference)	0.0093 (−0.1048 − 0.1235)	1.01 (0.90 − 1.13)	0.8725
OR costs			
EE versus SSS (reference)	−0.0212 (−0.1483 − 0.1059)	0.98 (0.86 − 1.11)	0.7435
EE versus SSH (reference)	−0.2130 (−0.3381 − −0.0879)	0.81 (0.71 − 0.92)	0.0009
SSH versus SSS (reference)	0.1918 (0.0887 − 0.2950)	1.21 (1.09 − 1.34)	0.0003
OR material costs			
EE versus SSS (reference)	−0.6457 (−1.0079 − −0.2836)	0.52 (0.36 − 0.75)	0.0005
EE versus SSH (reference)	−0.4307 (−0.7905 − −0.0710)	0.65 (0.45 − 0.93)	0.0191
SSH versus SSS (reference)	−0.2150 (−0.5134 − 0.0834)	0.81 (0.60 − 1.09)	0.1574

*EE* end-to-end anastomosis, *SSH* side-to-side handsewn anastomosis, *SSS* side-to-side stapled anastomosis, *CI* confidence interval, *OR* operating room

## Discussion

This retrospective single-center cohort study analyzed the costs of three different anastomotic techniques after right-sided colectomy during a period from January 2016 to December 2021. While the total costs did not show any significant differences (*p* = 0.1657), the end-to-end anastomosis was the most cost-effective in terms of OR costs (*p* < 0.0001) and OR material costs (*p* < 0.0001*)*. Regarding the short-term outcome, there were no significant discrepancies between the different groups with respect to postoperative (*p* = 0.1900) or case-related complications (*p* = 0.5032) and 30-day mortality (*p* = 0.8410).

In recent years, healthcare costs have continued to rise, leading to increased financial pressure on health care providers both globally as well as in Switzerland [14, 19]. As a result, cost-effectiveness has become a key area of focus, alongside advancements in technology and quality. Sheetz et al. (2018) showed in a study population with 428,799 patients who underwent a colectomy, that laparoscopy was associated with significantly lower medical expenditures than open procedures. They interpreted these differences mainly as the cause of lower complication rates in the laparoscopic group. However, they also highlighted that the surgeon’s expertise and skills in minimal-invasive surgery had a significant impact on postoperative complications and thereby the associated readmissions and follow-up care costs [20]. Although our hospital is moving towards minimally invasive methods, we deliberately included both minimally invasive and open right-sided colectomies in our study, as each was the safest method chosen by the surgeon. Additionally, we think that this variability approximately reflects the heterogeneity of performed procedures in other hospitals in Switzerland and especially in the rest of Europe, where minimal-invasive techniques might not yet have been fully established.

Moreover, in our study, no difference in costs was found between teaching procedures and standard operations. This could be attributed to the fact that even when the anastomosis was performed by a less experienced surgeon, it was supervised by a senior surgeon. Consequently, we consider right-sided colectomy an essential procedure for training younger surgeons in anastomotic techniques within a controlled and secure environment. Although the trend is moving increasingly towards minimally invasive techniques, including intracorporeal anastomosis, we think that a solid foundation in open surgery remains essential in surgical training to ensure safe alternative surgical techniques in cases where no minimally invasive procedure can be performed.

In our study, we demonstrated that the EE technique was significantly the most cost-effective in terms of both overall OR costs and OR material costs. We attribute this primarily to the fact that no stapler materials were used, unlike in the other two groups. However, no significant difference in total costs was observed. The EE group had the highest proportion of open procedures and the longest hospital stays, which we believe accounts for the balance in overall costs. It has already been demonstrated in several studies that laparoscopic techniques can lead to shorter hospital stays and lower costs due to faster recovery [13, 21]. In our data, we also observed a trend over time towards increased use of minimally invasive techniques and shorter hospital stays. Another factor contributing to the decreasing length of stay over time is the implementation of ERAS protocols, which were first developed in the 1990s. These protocols focus on improving short-term outcomes by minimizing surgical stress and enhancing recovery [22]. Zhuang et al. published a meta-analysis of five randomized clinical trials comparing open and laparoscopic surgery with concurrent use of ERAS protocols [23]. They demonstrated the benefits of laparoscopy over open surgery, particularly in terms of outcomes. The ERAS protocol also showed advantages regarding length of stay and short-term outcomes. However, the authors pointed out that the ERAS protocols used in the studies were not fully optimized.

As the adoption of minimally invasive techniques in right colectomy continues to expand, the Torino trial [12] investigated the costs in ileocolic anastomosis associated with intra- versus extracorporeal anastomosis techniques in a single-institution double blinded randomized controlled trial. A total of 140 patients were randomized either in an intracorporeal or extracorporeal anastomosis group before performing a laparoscopic right colectomy. In their study the intracorporeal anastomosis had higher mean overall costs (*p* = 0.704) with significantly higher costs in intraoperative costs (*p* < 0.001) and costs of surgical instruments (*p* < 0.001). The mean costs of room occupancy as well as postoperative and outpatient costs were comparable. The authors of this study identified as one of the limitations that in the extracorporeal group, both stapler and hand-sewn anastomosis were performed, whereas in the intracorporeal group, only stapler anastomosis was conducted. Since intracorporeal anastomosis had not yet been applied in right-sided colectomy at our hospital at the time of the study, we set out to compare the different reconstruction techniques. To the best of our knowledge, we are the first to conduct a cost analysis addressing this specific question.

As technology continues to evolve, robotics is playing an increasingly significant role in minimally invasive surgery. The three-dimensional vision and extended range of motion provided by robotic surgery enable skilled surgeons to perform more precise intra-abdominal work compared to laparoscopy. This is particularly beneficial for right-sided colectomies, where the intracorporeal anastomosis can be executed with greater precision. Several studies have shown that robotic surgery can lead to a faster postoperative recovery and reduced conversion rate [24, 25]. Despite the higher intraoperative costs, robotic-assisted surgery has the potential to reduce patient length of stay and may eventually decrease overall costs. During the study period, we performed 13 robotic-assisted cases. However, due to the small sample size, these cases were excluded from our analysis. Future studies should include robotic surgery, particularly focusing on intracorporeal anastomosis, to better understand its cost-effectiveness and clinical outcomes.

As a secondary endpoint, we examined morbidity and mortality, finding no significant differences between the groups. This aligns with the findings of other studies [11, 26], who also reported no significant differences in these outcomes. One important complication in colorectal surgery is anastomotic leak, which increases both, morbidity and mortality and can consequently lead to higher healthcare costs. According to the literature, anastomotic leak rates range from 1.2 to 7.4% [8, 27, 28]. A 2020 study, which analyzed 6548 right-sided colectomies performed in Australia and New Zealand over 12 years, reported an anastomotic leakage rate of 2.1% [27]. With an anastomotic leakage rate of 2.6%, we achieved favorable short-term outcomes, which can likely be attributed in part to the high proportion of elective procedures (72%).

Several limitations of this study must be acknowledged. Although this is a retrospective study, we were able to include a relatively large and thus representative sample size with nearly complete datasets compared to other cost analyses (such as the Torino study), allowing us to draw conclusions about the cost-effectiveness of the different types of anastomoses. We must note that the EE group was substantially smaller than the SSH and SSS groups and it had the highest proportion of open procedures and privately insured patients. The heterogeneity in the choice of anastomosis type is partly due to the lack of a defined standard in our hospital during the study period. However, this variability is consistent with existing literature, which does not identify an explicit gold standard for the choice of anastomosis technique [1, 11]. Due to the differing distribution of insurance classes across the groups, we focused on total costs rather than revenues to reduce potential bias. Another limitation is that our results might not apply in other countries regarding insurance system in terms of reimbursement and in different educational systems.

In conclusion, our analysis revealed that the various anastomosis techniques did not result in statistically significant differences in short-term outcomes or in overall costs. EE anastomosis was associated with the lowest OR costs without any disadvantages concerning postoperative complications and given a very low overall complication rate in this cohort. Therefore, we feel in the light of cost-constrained health care settings EE remains a viable option either in open or laparoscopic-assisted right-sided colectomies and should be part of every colorectal surgeon’s skill set. Nevertheless, considering the increasing prominence of minimally invasive techniques with consequently shorter hospitalization durations compared to open surgery, it is unlikely that EE will become the standard technique. This is further supported by our findings, which showed no overall cost benefit for EE in open surgeries. Therefore, future economic studies should focus on these emerging methods as they become more central to surgical practice.
